# Waste-Derived Nanoparticles: Synthesis Approaches, Environmental Applications, and Sustainability Considerations

**DOI:** 10.3389/fchem.2020.00782

**Published:** 2020-08-31

**Authors:** Sabah M. Abdelbasir, Kelli M. McCourt, Cindy M. Lee, Diana C. Vanegas

**Affiliations:** ^1^Central Metallurgical Research and Development Institute, Cairo, Egypt; ^2^Department of Environmental Engineering and Earth Sciences, Clemson University, Clemson, SC, United States; ^3^Department of Engineering and Science Education, Clemson University, Clemson, SC, United States; ^4^Interdisciplinary Group for Biotechnological Innovation and Ecosocial Change-BioNovo, Universidad del Valle, Cali, Colombia

**Keywords:** carbon nanoparticles, metal nanoparticles, e-waste, plastic waste, industrial waste, nanoparticle-enabled technologies

## Abstract

For the past few decades, a plethora of nanoparticles have been produced through various methods and utilized to advance technologies for environmental applications, including water treatment, detection of persistent pollutants, and soil/water remediation, amongst many others. The field of materials science and engineering is increasingly interested in increasing the sustainability of the processes involved in the production of nanoparticles, which motivates the exploration of alternative inputs for nanoparticle production as well as the implementation of green synthesis techniques. Herein, we start by overviewing the general aspects of nanoparticle synthesis from industrial, electric/electronic, and plastic waste. We expand on critical aspects of waste identification as a viable input for the treatment and recovery of metal- and carbon-based nanoparticles. We follow-up by discussing different governing mechanisms involved in the production of nanoparticles, and point to potential inferences throughout the synthesis processes. Next, we provide some examples of waste-derived nanoparticles utilized in a proof-of-concept demonstration of technologies for applications in water quality and safety. We conclude by discussing current challenges from the toxicological and life-cycle perspectives that must be taken into consideration before scale-up manufacturing and implementation of waste-derived nanoparticles.

## Introduction

According to the Environment Program of the United Nations, nearly 11.2 billion tons of solid waste is generated every year, which is a significant source of environmental degradation and negative health impacts, particularly in low-income countries, where more than 90% of waste is openly dumped or burned (UNEP, 2020). In Southeast Asian countries with insufficient waste management capacity, such as Vietnam, Malaysia, the Philippines, and Thailand, waste-related issues are further exacerbated by the massive amounts of plastic, electric, and electronic waste imported from industrialized countries (Kaza et al., 2018; Dell, 2019; Sukanan, 2020). This situation has motivated the development, implementation, and strengthening of different policy strategies including (i) international environmental agreements such as the Basel Convention on the control of transboundary movements of hazardous wastes and their disposal (Basel Action Network, 2019), (ii) source reduction, or complete bans on single-use plastics (UNEP, 2018), and (iii) shifts in political agendas toward the adoption of circular economy models for meeting environmental and resource-oriented goals in several countries (Bourguignon, 2014; Berg et al., 2018). However, there are significant technical, economic, environmental, and social challenges for realizing closed-loop management of engineered materials on a large scale. For example, many plastic and metal recycling processes involve the use of hazardous substances for extraction and purification, which results in new health risks for humans and the environment (Kral et al., 2019). Besides, the practical viability of strategies for recycling and repurposing materials is directly linked to the economic value of the end-products. Therefore, using “clean manufacturing” processes to yield “value-added” commodities from waste materials emerges as a desirable synergy for achieving both circularity and sustainability goals (Ferronato and Torretta, 2019).

Over the past few years, a plethora of engineered nanomaterials have demonstrated great promise for advanced applications ranging from energy transformation and storage (Hussein, 2015; Verma et al., 2016; Sonawane et al., 2017; Yang et al., 2019), pollution monitoring (Baruah and Dutta, 2009), intelligent packaging (Pereira de Abreu et al., 2012), precision agriculture and controlled delivery of food ingredients (Bindraban et al., 2015; Peters et al., 2016), membrane technology (Goh et al., 2015); water treatment (Westerhoff et al., 2013); drug delivery and diagnostics (Turcheniuk and Mochalin, 2017), bone and tissue engineering (Shadjou and Hasanzadeh, 2016), amongst others. In an effort to reduce negative environmental impacts and health risks associated with the production, use, and disposal of novel nanomaterials, the fields of materials engineering and nanotechnology are increasingly concerned with sustainability approaches, frameworks, and metrics (Dhingra et al., 2010; Mata et al., 2015; Falinski et al., 2018). For example, a Boolean search of the keywords “green synthesis” AND “nanomaterials” on the academic engine *Web of Science* (Clarivate™) returns a record count of 569 articles published on 2019 alone, which is 3-fold higher number than the year 2015, and 12-fold higher than the year 2009. This growing trend is expected to continue over the next few years, consistent with global trends on sustainable development.

**Figure 1:**
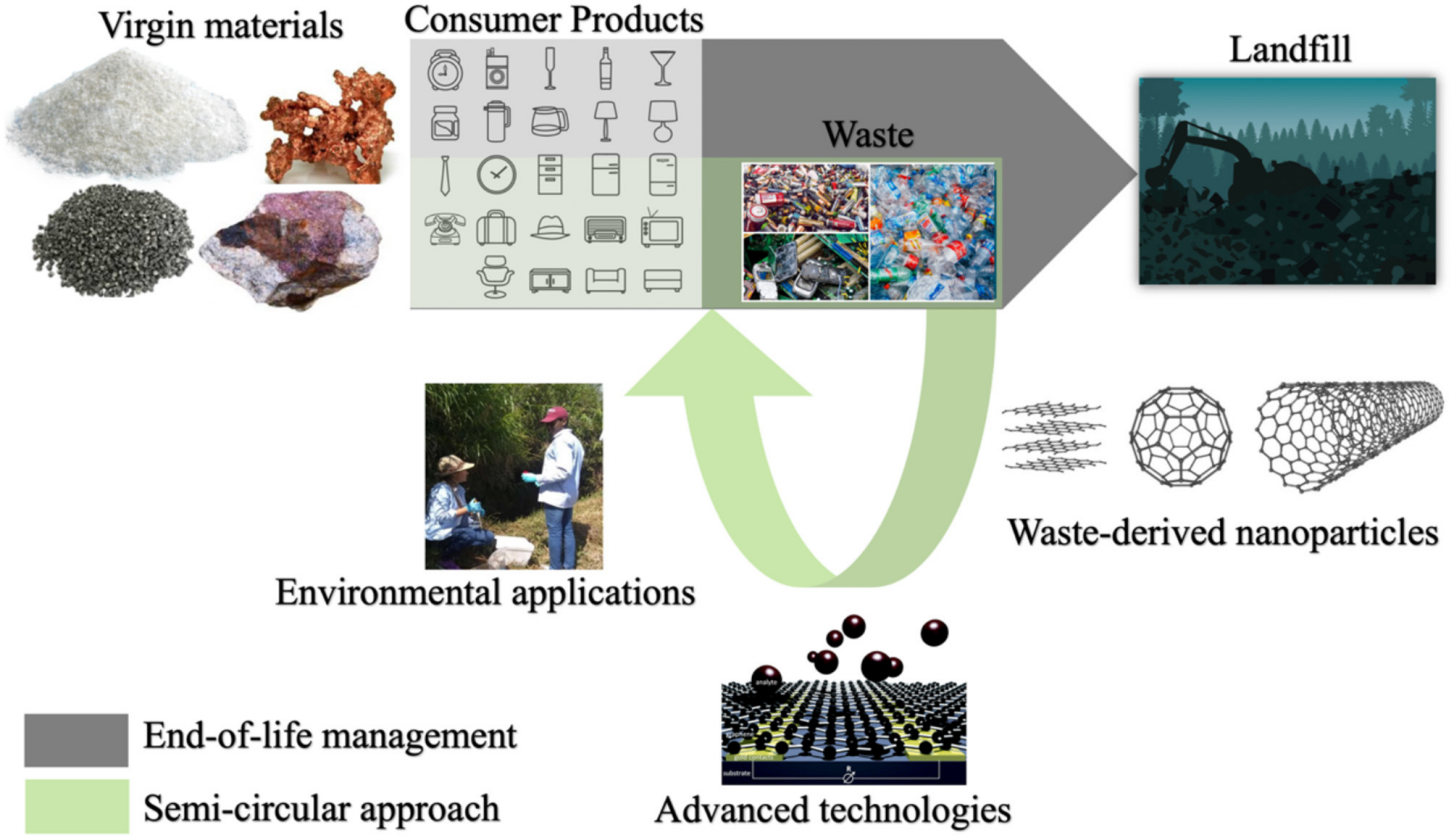
Schematic representation of the concept discussed in the review article. industrial, electric, electronic and plastic wastes are suitable inputs for sustainable production of engineered nanoparticles, which can be further used in the development of advanced technologies for environmental applications. Thus, disrupting the linear *end-of-life* approach to waste management and increasing sustainability by *closing the loop* with the fabrication of value-added materials and technologies from waste. This schematic was created using public domain images obtained from a open source database.

Herein, we provide an overview of the current status, challenges, and future directions for the utilization of industrial wastes (e.g., large batteries, rubber tires, wastewater), e-waste (e.g., copper cables, printed circuit boards, electronic equipment), and plastic wastes (e.g., polyethylene, polypropylene, polyvinyl alcohol) as suitable inputs for production of nanoparticles (NPs) as added-value products. In addition, we review some proof-of-concept examples for the incorporation of waste-derived NPs in advanced technologies for pollution monitoring, treatment, and remediation of water (Figure 1). We conclude with some remarks from the sustainability viewpoint, that emphasize the critical aspects necessary for scale-up manufacturing and deployment of waste-derived nanomaterials.

## Waste as Starting Materials for the Production of Nanoparticles

Depending on the source of emission, highly problematic waste materials produced in massive amounts can be classified into wastes generated by industries, and wastes generated by consumers. Both industrial and consumer wastes can be utilized as inputs for processes that yield value-added products. From the industrial sector, large batteries, rubber tires, wastewater, and biosolids are prominent sources of carbon, lead, zinc, copper, and palladium. End-of-life consumer products result in overwhelming amounts of e-waste and plastic waste. E-waste can be a significant source of recoverable precious and semi-precious metals. Depending on the type of polymer and the degree of purity, plastic waste can be either recycled and reused as packaging material or, it can be used as raw material for other applications, including the production of construction materials, paper, fiber composites, new polymers, and carbon nanoparticles. In this section, we examine the recent research on the mechanisms and processes used for the recovery of valuable components from the different waste streams.

### Industrial Waste

Up to this point in time, several industrial wastes such as batteries, tires, wastewater, and sludges have been studied as potential low-cost and ubiquitous starting materials for nanoparticles synthesis (Samaddar et al., 2018).

#### Lead Batteries

Over 50 million units of waste lead batteries are produced annually by China alone; considering the toxicological effects of lead exposure, along with its bioaccumulation capacity, recycling these batteries is a responsible approach while avoiding ecological and public health problems (Zhou et al., 2017). Lead pastes are made of metal oxide, dioxide, and sulfate forms that are difficult to recover and reuse because of their insolubility. However, pyrometallurgical processes can be employed to retrieve lead from spent lead-acid battery paste. For example, Zhou et al. (2017) designed a synthesis technique to make Pb nanopowder from simulated spent lead paste by the pyrolytic route. The authors produced submicron particles of lead oxide by a thermal process coadjuvated by poly (N-vinyl-2- pyrrolidone), which helps decrease the particle size (Zhou et al., 2017). While the technique overcomes difficulties associated with the insolubility of lead paste in aqueous media, pyrometallurgical processes may have unfavorable consequences due to the high energy requirements as well as the undesirable emissions of air pollutants such as sulfur dioxide and lead dust.

#### Zinc-Manganese Batteries

Similarly, a large percent of transportable batteries in industrialized countries are made of zinc-manganese (Zn-Mn) composites. China alone produces more than 15 billion units every year (Xiang et al., 2015). Exposure to toxic concentrations of Zn can result in impaired calcium uptake and metabolic deficiencies in humans and other organisms (Valko et al., 2005). Thus, recovering Zn from recycled Zn-Mn batteries is a convenient approach for enabling reuse of the metal in other applications while limiting its uncontrolled dissemination in the environment. Synthesis of Zn nanomaterials such as NPs, nanofibers, and flaky NPs using recycled Zn-Mn batteries as starting material, has been actively investigated in recent years. Xiang et al. (2015) demonstrated a facile and highly efficient method for separating zinc from spent batteries. Samples of recycled zinc-cathodes were put in a corundum crucible and subjected to a vacuum pressure of 1 Pa, heating temperature of 800°C, and condensing temperature 200°C. The simple technique can attain zinc separation efficiency up to 99.68%. By following the separation process with a nitrogen gas flow into the heating chamber, structured nanoparticles can be obtained with different morphologies including hexagonal prisms, fibers, and sheets (Xiang et al., 2015).

#### Rubber Tires

Worldwide, roughly 1,000 million scrap tires are generated every year (GoldsteinResearch, 2020). A significant portion is landfilled or openly burned, contributing to air pollution and environmental degradation (Moghaddasi et al., 2013). For that reason, the disposal of scrap rubber in landfills is banned in most developed countries (Turgut and Yesilata, 2008). Thus, there is interest in finding sustainable solutions for managing rubber tire waste. Generally, tires are composed of 45–47% natural rubber, 21–22% carbon black, 12–25% metals, 5–10% textile, 6–7% additives and 1–2% zinc, ~1% sulfur (Evans and Evans, 2006; Samaddar et al., 2018). The synthesis of Zn NPs from used tires constitutes a suitable alternative with value-added potential. Moghaddasi et al. (2013) obtained NPs from recycled tires by applying ball milling for five consecutive hours. The method yielded rubber ash particles smaller than 500 nm; when aided with silicon waste, the particle size was further reduced to <50 nm. The effectiveness of rubber ash and ground tire rubber particles as a zinc source for plant nutrition was comparable to a commercial ZnSO_4_ fertilizer (Moghaddasi et al., 2013).

As waste tires are composed, principally, of carbon (representing about 81.2 wt%) they are considered a promising source of carbon for various potential applications. Maroufi et al. (2017) synthesized high-value carbon nanoparticles (CNPs) from waste tire rubber (WTR), using a high-temperature approach. The transformation of WTRs was carried out at 1,550°C over various reaction times (5 s to 20 min). The total energy utilization for the process was acceptable as the decomposition of WTR and the formation of CNPs occurs quickly, and completely. Within 5 min, all the impurities, such as sulfur, zinc, and oxygen, have been removed (Maroufi et al., 2017). Another facile and low-cost method for the preparation of carbon black nanoparticles from waste tires was reported by Gómez-Hernández et al. (2019). Briefly, thermal transformation under the high temperature (1,000°C) and self-pressurized conditions produced carbon nanoparticles (~22 nm) with high yield (about 81%) under the optimized conditions. The chain-like agglomerated nanoparticles showed good thermal stability and conductivity and the chemical analysis indicated that it was partly oxidized (C, 84.9%; O, 4.9%; S, 10.21%) (Gómez-Hernández et al., 2019). Many studies have been reported advising to choose an unconventional hydrocarbon waste as low-cost starting materials such as tires and plastics for the synthesis of CNTs (Sathiskumar and Karthikeyan, 2019). Multiwalled carbon nanotubes were synthesized from low boiling point hydrocarbon tire pyrolysis oil derived from waste tire material mixed with ferrocene as the catalyst on a quartz substrate with a flow rate of 20 ml/min at 950°C (Parasuram et al., 2018).

#### Wastewater and Bio-sludge

Wastewater effluents and industrial sludges have been identified as rich sources of valuable metals and metal oxides; therefore, these waste effluents have been extensively studied for the recovery of materials such as PbO, Mo, Ni, Co, Cu, Ag, SiO_2_, rare earth elements, and Pt. While the concentration of trace elements is highly variable among waste effluents, there are associations with the specific activities performed in the emitting source, as well as the mineralogical diversity found within a single effluent source. For example, the U.S. Geological Survey has identified waste effluents from Zn ore mining as major sources of Ga and In. Through wastewater and biosolid mining, Ga and In could be recovered and used for the fabrication of integrated circuits, light-emitting diodes, photodetectors, semiconductors, and solar cells (Smith et al., 2015). An elemental analysis of waste stream samples (e.g., mining influenced waters) from different historical metal mining sites found concentrations of Ag (Mdn. ~10 ppm), Au (Mdn. ~0.3 ppm), Pd (Mdn. ~30 ppm), Pt (Mdn. ~1 ppm), Re (Mdn. ~ 0.01 ppm), Os (Mdn. ~0.02 ppm), Ir (Mdn. ~0.001 ppm), La (Mdn. ~20 ppm), Ce (Mdn. ~40 ppm), Nd (Mdn. ~20 ppm), Sm (Mdn. ~4 ppm), Gd (Mdn. ~4 ppm), Yb (Mdn. ~2 ppm), Th (Mdn. ~4 ppm), Zn (Mdn. ~1,000 ppm), Cu (Mdn. ~150 ppm), Ga (Mdn. ~15 ppm), In (Mdn. ~ 1 ppm), Sb (Mdn. ~10 ppm), and Te (Mdn. ~1.5 ppm). Although the recovery of some of these metals may not be economically attractive by itself, the removal and recovery of trace elements from waste streams may still offset treatment and disposal costs and reduce environmental and public health liability (Smith et al., 2015).

Urbina et al. (2019) demonstrated an innovative approach for metal recovery from aqueous waste such as those found in bioremediation or biomining processes. The method uses metal-binding peptides to functionalize fungal mycelia. *In silico* models have been developed for predicting binding affinity between metals and natural ligand-binding motifs. These models are useful because they enable the prediction of binding parameters based on open access protein databases (e.g., The Protein Data Bank https://www.rcsb.org) to describe the geometric features a cognate metal in a metalloprotein. As a proof-of-concept, the authors achieved binding affinity and specificity for Cu that show a high correlation between the natural motifs and those derived *in silico* (Urbina et al., 2019). Another study identified different morphologies of silica nano-fillers (100–500 nm) present in polymer waste (Tran et al., 2017). Various metals have also been recovered from pickling wastes, spent catalysts, furnace slag, fly ash, e-waste, and metalized plastic waste (Khaloo et al., 2013; Bennett et al., 2016; Chaukura et al., 2016; Chen et al., 2016; El-Nasr et al., 2020; Elsayed et al., 2020). Aside from being a good source for the recovery of metals, various industrial effluents and bio-wastes have been used for the synthesis of carbon-based nanomaterials (Deng et al., 2016; Zhang et al., 2016). Deng et al. provided a comprehensive review highlighting the current status and prospects of green synthesis of carbon nanocomposites from bio-sludges and industrial wastes through microbiologically driven pathways (Deng et al., 2016).

### Electric and Electronic Wastes

The overwhelming generation of end-of-life electric and electronic products and equipment (e-waste or WEEE) in the United States and Europe is causing severe environmental and public health issues in the e-waste receiving nations, which are primarily located in West Africa and Asia (Baldé et al., 2017; Tansel, 2017). For the past decades, there has been a steady increase in the development of efficient recycling techniques for e-waste led by the US and China, especially with regards to the recovery of metallic components (Bhat et al., 2012; Wibowo et al., 2014; Barletta et al., 2015; Park et al., 2017; Zeng et al., 2017). However, e-waste recycling is increasingly challenging because electronic devices are made of complex mixtures of various metals, plastics, glass, and even newly engineered nanomaterials (Abdelbasir et al., 2018b,c). Moreover, electronic components are continually being miniaturized, which diminishes the efficiency of conventional mechanical separation processes available in developing countries (Baldé et al., 2017). Despite the practical difficulties, the high economic value of precious metals, such as gold, silver, and palladium, continues to drive e-waste mining.

As shown in Table 1, Printed circuit boards and mobile phones contain the highest amounts of precious metals and a considerable portion of base metals. Printed circuit boards (PCBs) are an integral component of all electronics. On average, PCBs represent 3% of the total mass of waste electric and electronic equipment, but in the case of small appliances, it can account for almost 8% of the weight (Dalrymple et al., 2007; Luda, 2011; Abdelbasir et al., 2018c). In 2014 the value of metals in e-waste was roughly estimated at 52 billion U.S. dollars (Xu et al., 2016). The estimated overall composition of PCBs is 40% metals, 30% organics, and 30% persistent noxious materials (Abdelbasir et al., 2018b). Detailed elemental analysis of polymers present in PCB has revealed the following composition: 5.52 wt% C, 2.18 wt% H, 0.73 wt% N, and 7.86 wt% Br (Blazsó et al., 2002). Copper represents the highest amount of metals in PCBs, and it has the second-highest pecuniary value. The organics are generally made of a variety of polymers such as polyvinyl chloride (PVC), polycarbonate (PC), acrylonitrile butadiene styrene (ABS), polytetrafluoroethylene (PTFE), polyethylene (PE), and polypropylene (PP) (Stevens and Goosey, 2009). The toxicity of the materials found in PCBs is a recurrent concern in the characterization and treatment of waste PCBs. The lead-tin soldering material and the brominated flame retardants (BFRs) contained in PCBs are the most harmful constituents. Additionally, copper catalyzes the formation of dioxins when the flame retardants are incinerated.

**Table 1:** Composition of metals in various e-waste scraps.

**Type of e-waste**	**Weight (%)**					Weight^*^ **(ppm)**		
	**Cu**	**Fe**	**Al**	**Ni**	**Pb**	**Ag**	**Au**	**Pd**
Printed circuit board	20	6	4	1	2.5	1,000	250	90
Mobile phone	13	5	1	0.1	0.3	1,380	350	210
TV board	10	28	10	0.3	1.0	280	20	10
Portable audio	21	23	1.0	0.03	0.14	150	10	4
DVD player	5	62	2	0.05	0.3	115	15	4
Calculator	3	4	5	0.5	0.1	260	50	5

*This table was partially reproduced from Hsu et al. (2019) with republication license provided by the Royal Society of Chemistry (license ID: 1034053-1) (Hsu et al., 2019)*.

* *Minor components (<0.03 wt.%)*.

Conventional industrial waste management processes involve transport of e-waste over long distances, to only recover a small fraction of materials; primarily precious metals such as Au and Ag, and semi-precious metals, like Cu, Ni, Al, Zn, Pb, and Sn, leaving significant amounts of residual waste and toxic components (Mankhand et al., 2012). Generally, industrial recycling approaches start with energy-intensive mechanical operations such as bulk crushing, grinding, and separation based on density or magnetic properties (Long et al., 2010); chemical treatments are later applied for extraction and purification of copper and other precious metals (Quan et al., 2010; Mankhand et al., 2012).

Overall, the metal content in e-waste is much higher than in land-mined ores (Li et al., 2007; Duan et al., 2009; Zhao et al., 2012). For example, e-waste has a weight composition of 10–20% Cu, whereas the maximum concentration of Cu in virgin ore is 3%. Thus, recovering metallic elements from e-waste, rather than mining for ores in natural deposits, would significantly cut the environmental impacts associated with both the mining of metals and the accumulation of e-waste. Enormous energy savings have been estimated from the recovery of metals from e-waste in contrast with the conventional extraction from mined ores. Specific examples of comparative cost-benefits are 95% for Al, 85% for Cu, 75% for Zn, and 90% for Ni (Cui and Forssberg, 2003; Shokri et al., 2017). Conventional approaches for recovering metals from e-waste begin with the conversion of the metallic components found in e-waste to oxide or sulfite forms, followed by the iterative raising of the pure form of the elements. Recently, efficient processes such as pyro- and hydro- metallurgy of e-waste captured significant attention. In pyrometallurgy, crushed e-waste is placed into a molten bath where the plastic degrades while releasing energy, and the refractories are ejected as slag. This slag is subsequently processed for the extraction of base or precious metals (Cayumil et al., 2014). Alternatively, hydrometallurgy processing is more predictable and controllable, but also very costly since it requires recovery and treatment of spent liquids and preparation of fresh solutions. Furthermore, the presence of chlorides in hydrometallurgically-treated e-waste is likely detrimental to the environment (Mankhand et al., 2012).

While the high yields of precious and semiprecious metals from WEEE provide the economic incentive for recycling it, there is still a need for more efficient and sustainable approaches to WEEE management that use a more substantial fraction of the waste materials (not just the metal parts) and results in the generation of value-added products (Abdelbasir et al., 2018c).

### Plastic Wastes

The broad utilization of non-biodegradable plastics has arguably brought about the most significant and problematic class of waste materials in the world. Plastic polymers have been intensely used for about a century to fabricate an immense number of products. In fact, the production of synthetic plastics has increased vastly during the last decades (Gu and Ozbakkaloglu, 2016). Traditional plastics were designed to be durable and withstand variations in external environmental conditions, which is precisely the reason why they persist and accumulate in the environment (Pol and Thiyagarajan, 2010). On a global scale, plastics are now the major component in solid waste (Ritchie and Roser, 2020). The generation of plastic wastes (PW) accounts for over 150 million metric tons per year (Aboulkas et al., 2010).

Plastic debris and microplastics have been found in alarming concentrations in the ocean. These particles produce negative impacts not only on aquatic life and birds but also on humans via contamination of the trophic chain (Bouwmeester et al., 2015). Aside from the problems associated with their slow degradation, PWs are harmful because they contain pigments with several highly noxious trace elements that can migrate out of the polymeric matrix and into the environment (Gondal and Siddiqui, 2007). As a result, environmental pollution from synthetic PWs is now considered one of the most devastating and likely irreversible effects of contemporary anthropogenic activities (Zheng et al., 2005).

In many countries, separation at source (i.e., segregation of materials at the point of disposal) and recycling are main collective efforts initiated at the household level, which are aimed at reducing the need for producing new plastics from virgin materials and fossil fuels. Main synthetic polymers in household items include polystyrene (PS), polypropylene (PP), polyethylene terephthalate (PET), high-density polyethylene (HDPE), low-density polyethylene (LDPE), linear low-density polyethylene (LLDPE), polyvinyl chloride (PVC) and nylon. Table 2 depicts the properties of common plastic polymers. Considering the vast amount of solid waste generated worldwide, stakeholders are looking for avenues to use PWs as starting material for energy recovery or valued-added products manufacturing (Ackerman, 2000). From the thermodynamic perspective, energy recovery is less favorable as the energy content of plastic (42.6 MJ/l) is at least an order of magnitude lower than conventional fuels such as heating oil (443.5 MJ/kg) (Kumar et al., 2011; Banu et al., 2020). Additionally, using PW as an energy source can be seriously detrimental for public health since harmful dioxins are released during PW burning. Finally, the concept of using PWs as an alternative feedstock to generate syn-gas or oil for the later conversion into liquid fuels such as diesel and gasoline may incentivize the unnecessary perpetuation of dependence on carbon fuels, which ultimately leads to the release of greenhouse gases into the atmosphere, and all its encompassing effects on the planet.

**Table 2:** Properties and structure of polymers commonly found in plastic waste.

**Plastic**	**Molecular weight (g/mol)**	**Density, (g cm^**−3**^)**	**Chemical formula**	**Structure**	**Crystallinity**
Polystyrene (PS)	104.1 (of repeat unit)	1.04–1.11	(C_8_H_8_)_n_	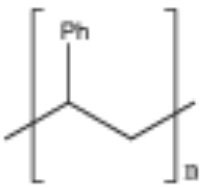	Highly amorphous (atactic), Highly crystalline (syndiotactic)
Polyvinylchloride (PVC)	62.50 (of repeat unit)	1.10–1.45	(C_2_H_3_Cl)_n_	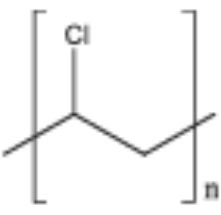	Highly amorphous
polyethylene terephthalate (PET)	192.17 (of repeat unit)	1.38–1.40	(C_10_H_8_O_4_)_n_	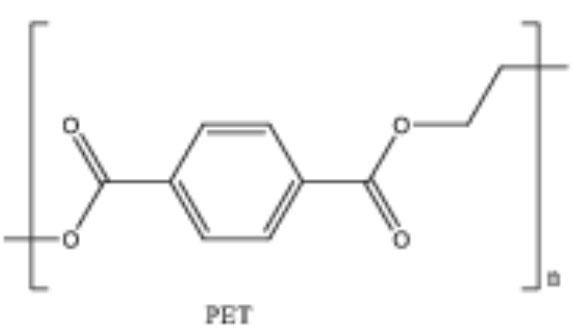	Semi crystalline
Polypropylene (PP)	42.08 (of repeat unit)	0.855, amorphous 0.946, crystalline	(C_3_H_6_)_n_	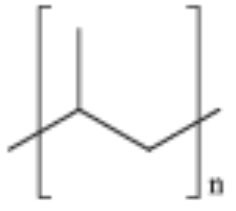	Highly crystalline (isotactic), Highly amorphous (atactic)
High-density polyethylene (HDPE)	10^3^–10^7^	0.93–0.97	(C_2_H_4_)_n_	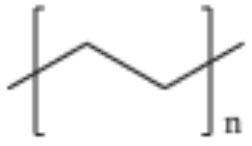	High crystalline and low amorphous
Low-density polyethylene (LDPE)	8.9 × 10^4^ to 4.7 × 10^5^	0.910–0.940	(C_2_H_4_)_n_	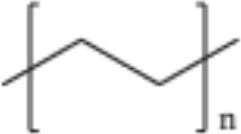	Low crystalline and high amorphous

On the other hand, turning PW into useful products is particularly challenging because of the chemical heterogenicity in the different waste streams. For instance, many packaging materials are made of compact composites of several thin layers of different polymers and often are lined with aluminum. These composite plastics have to be landfilled because they are difficult to separate by conventional mechanical and/or chemical recovery processes (Al-Salem et al., 2009; Astrup et al., 2009; Singh et al., 2017). Recent approaches for valorizing mixed PWs include mixing them with materials such as wood, cement, and ash to turn them into construction materials (Sardot et al., 2012; Sofi et al., 2019), printable paper-like composites (Fan et al., 2020), fiber composites (Keskisaari and Kärki, 2017), new polymers (Rahimi and Garciá, 2017), and polar waxes (Marek et al., 2015). Nanomaterials have also been prepared from single-polymer PW. For example, ultrafine nano-channeled carbon tubes and multi-walled carbon nanotubes (MWCNTs) have been synthesized from polyethylene terephthalate waste using an arc discharge method (Berkmans et al., 2014). Table 3 summarizes the advantages and challenges of some of the potential value-added applications of PWs.

**Table 3:** Potential value-added applications for recycled plastics.

**Recycled plastics**	**Value-added applications**	**Expected advantages**	**Foreseen challenges**	**References**
PET, PP, PS, LDPE, HDPE, PVC	Production of liquid fuels.	Reduced need for oil extraction from natural reserves.	High cost associated with complex thermochemical processes. Generation of hazardous byproducts.	Kumar et al., 2011; Banu et al., 2020
PP, LDPE, PS	Production of construction materials.	Possibility of utilizing mixed plastics.	Requires ultrafine grinding and thorough mixing to reduce variability in thermal and mechanical properties.	Sardot et al., 2012; Sofi et al., 2019
LLDPE	Production of printable paper.	High affinity with ink, hydrophobicity, and durability under harsh conditions.	Involves the use of hazardous organic solvents.	Fan et al., 2020
PVC, PET, PP	Production of fibers.	Reduced plastic waste volumes disposed to landfills.	Impurities and contamination potentially present in the recycled materials produce significant variations in the properties of the fibers.	Keskisaari and Kärki, 2017
LDPE, PET, HDPE, PVC, PS	Production of new polymers.	Reduced dependence on petrochemicals for plastic production. Catalytic methods for chemical recycling of polymers are less energy intensive than thermolysis methods.	Isolation and purification of monomers from chemically treated recycled polymers can be expensive. The mechanical properties of the newly synthesized polymers may not meet the conventional requirements.	Rahimi and Garciá, 2017
PP	Production of polar waxes	Facile 2-step process. Requires low-toxic solvents that can be recirculated into the process. The rheological properties of the obtained waxes are similar to the commercial ones.	Overtime, wax deposition may affect the equipment required for the process.	Marek et al., 2015
PP, PET	Production of carbon nanoparticles	Attractive for applications in a plethora of advance technologies.	Toxicity concerns; particularly in occupational settings where the nanoparticles are manufactured and handled in large quantities.	Bazargan and McKay, 2012; Berkmans et al., 2014; Zhuo and Levendis, 2014; Deng et al., 2016

In summary, the depicted mechanisms show promising potential for reducing the amount of landfilled materials and noxious elements dispersed in the environment. It is worth noting that the majority of the reviewed processes are still in the early stages of research and development, thus the economics of scale have not yet been fully determined. Nonetheless, the proposed processes serve as a proof-of-concept demonstration of ways to divert from the business-as-usual approach to waste management. Perhaps, offsetting treatment and disposal costs, and reducing environmental and public health liabilities may be sufficient incentives to promote these alternative approaches.

## Synthesis of Nanoparticles from Recycled Materials

### Metal and Metal Oxide Nanoparticles

Various procedures have been used for the synthesis of several types of nanomaterials from wastes after suitable pretreatment either physically or chemically or combinations of both. The most common physical pretreatment methods are grinding and milling. Chemical pretreatment is applied to separate any contaminants present in the waste sample by heating or treating with reagents such as strong acids (e.g., H_2_SO_4_, HNO_3_, HCl) (Samaddar et al., 2018).

The most popular synthesis method for metallic NPs is reduction with sodium borohydride. Generally, the sodium borohydride solution is freshly prepared and rapidly added to the solution of waste materials. NPs produced via reaction are washed repetitively with ultrapure water and absolute alcohol for the removal of excess sodium borohydride. This method was implemented by Fang et al. to prepare Fe NPs from steel pickling waste materials released from a steel plant (Fang et al., 2011).

Another important and widely used method is the solvent thermal method which involves chemical reactions in solvents contained in closed autoclave reactor and heated to a critical temperature (Dubin et al., 2010). When water is used as a solvent the process is known as “hydrothermal.” As an example, hematite NPs (α-Fe_2_O_3_) were synthesized via a solvent thermal method using ethanol as a solvent and heating the solution of the starting materials mixture (FeCl_3_, and NaOH) at 150°C for 2 h to gain NPs (Tang et al., 2011). Another technique that uses voltage to promote chemical reactions in aqueous solutions for the synthesis of nanoparticles is electrodeposition. This method has been applied to produce nanowires, nanoporous materials, and nanocylinders. In addition to these mentioned techniques, green synthesis methods based on the use of environmentally friendly and biocompatible materials such as plants and microorganisms have recently arisen (Jeevanandam et al., 2016).

**Figure 2:**
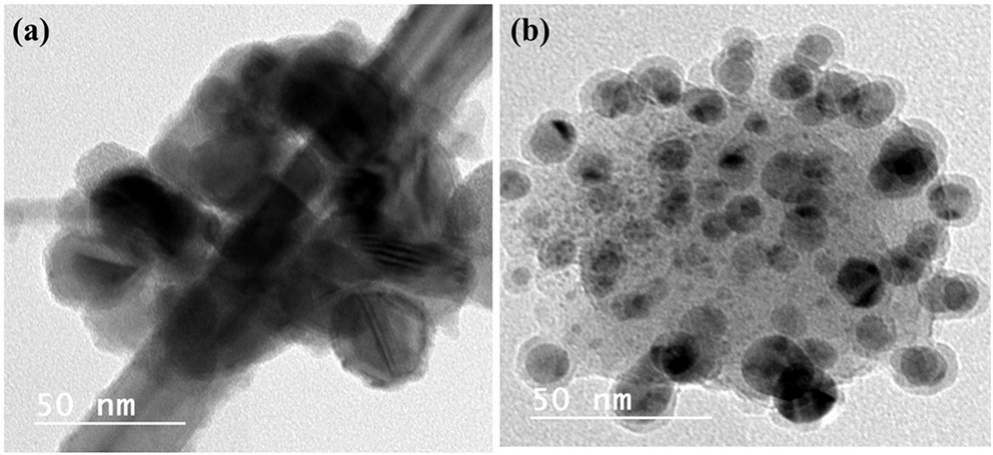
High resolution TEM images of CuNPs obtained from WPCBs. These NPs were prepared from **(a)** ammoniacal ammonium chloride and **(b)** ammoniacal ammonium carbonate leachant by ascorbic acid in presence of cetyltrimethylammonium bromide (CTAB) as modifier. These images were reproduced from Seif El-Nasr et al. (2020) under the reproduction license number 4854300796766 provided by Elsevier and Copyright Clearance Center.

Metal nanoparticles (e.g., Pb, Hg, Cu, Fe, Au, Ag, Pd, Pt, and Rh) and polymers can be recovered from electronic wastes such as computer circuit boards, cellular phones, laptops, automobiles, and supercapacitors (Xiu and Zhang, 2012; Singh and Lee, 2016; Vermisoglou et al., 2016). To date, most efforts have focused on the recovery of metal nanoparticles from spent batteries. However, other forms of e-waste are also good sources of a variety of valuable materials. For example, Cu nanoparticles were prepared from acidic CuCl_2_ waste etchants generated in the manufacturing process for printed circuit boards by microemulsion processes (Mdlovu et al., 2018). Likewise, aqueous solutions of CuSO_4_ from PCBs were chemically reduced to create organically stabilized Cu nanoparticles (Tatariants et al., 2018). Waste printed circuit boards (WPCBs) have also been recycled to obtain metals such as Cu, Pb, Fe, Au, and Hg (Calgaro et al., 2015; Chen et al., 2015a,b; Chu et al., 2015; Fogarasi et al., 2015; Hadi et al., 2015; Cui and Anderson, 2016). The metals recovered from WPCBs can be used to create nanoparticles. Cu from WPCBs has acted as a starting material in the synthesis of Cu nanoparticles (Yousef et al., 2018; El-Nasr et al., 2020). Figure 2 portrays the morphological features of Cu nanoparticles obtained from WPCBs using the approach by El-Nasr et al. (2020).

WPCBs can also be used to create nanoparticles directly. Cu_2_O/TiO_2_ photocatalysts were recovered using electrokinetic processes (Xiu and Zhang, 2009). Cu_2_O nanoparticles of different sizes were also created from WPCBs using supercritical water oxidation methods (SCWO) and electrokinetic processes (Xiu and Zhang, 2012). Copper–tin (Cu–Sn) nanoparticles were fabricated from the WPCBs of spent computers using selective thermal transformation while also separating toxic lead (Pb) and antimony (Sb) (Shokri et al., 2017). Lead (Pb) nanoparticles were synthesized from solders of WPCBs via evaporation under vacuum with forced flow inert gas condensation (Zhan et al., 2016). Various other forms of e-waste can be used to create nanoparticles such as purified carbon nanotubes, Cu_2_O nanoparticles, and Cu_2_O/TiO_2_ catalysts. For example, Ag was completely recovered from incinerated organic solar cells (Søndergaard et al., 2016).

**Figure 3:**
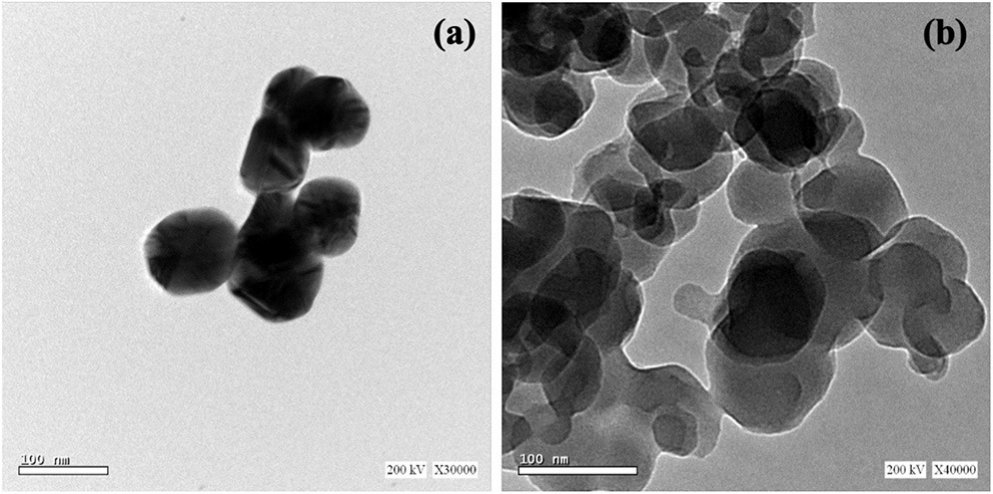
TEM images of **(a)** Ag and **(b)** Cu NPs synthesized from metalized plastic waste. The NPs were prepared with leachant solution of metalized acrylonitrile butadiene styrene (ABS) using ascorbic acid as reductant and chitosan as stabilizer according to the methods described by Elsayed et al. (2020). These original images were provided by the authors of the manuscript and have not been previously published.

Wastes other than e-wastes can also be utilized. Ag, Cu, and bimetallic Ag/Cu nanoparticles were synthesized from a leachate solution (Figure 3). This solution was derived from a solution of leached metalized acrylonitrile butadiene styrene (ABS) from plastic wastes combined with nitric acid and ascorbic acid in the presence of chitosan at 60°C (Elsayed et al., 2020).

**Figure 4:**
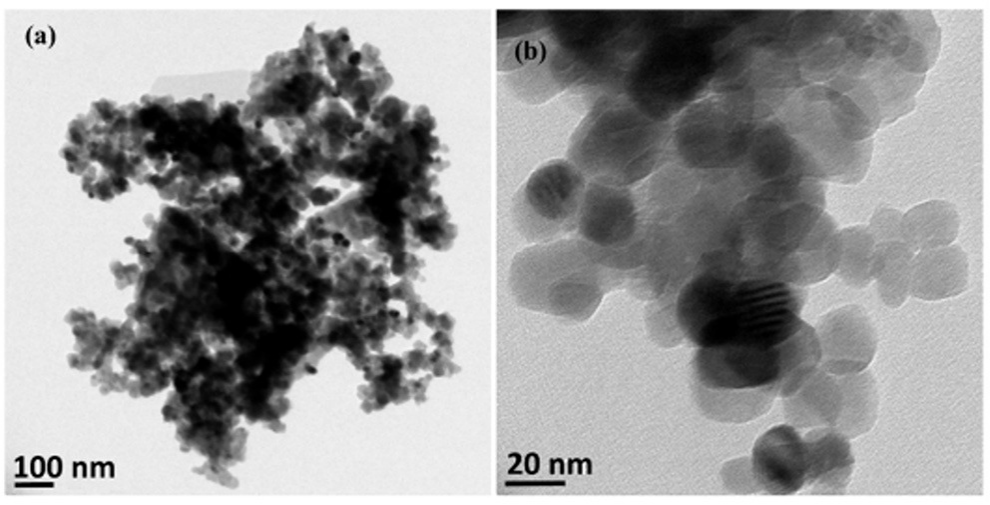
Low resolution **(a)** and high resolution **(b)** TEM images of ZnO NPs recovered from spent Zn-C batteries via thermal route. This figure was reproduced from Farzana et al. (2018) under the terms and conditions of the Creative Commons Attribution (CC BY) license (http://creativecommons.org/licenses/by/4.0/).

Researchers also utilize methods to reuse nanoparticles. For example, functionalized nanoparticles were successfully recycled to capture toxins from spiked blood plasma samples by applying a glycine buffer to free up the nanoparticles after their initial use (Hassanain et al., 2017). In another study, zinc oxide (ZnO) nanoparticles were synthesized from spent Zn-C battery via thermal technique at 900°C under an argon atmosphere using a horizontal quartz tube furnace. The produced nanoparticles were spherically shaped within a size range of 50 nm (Figure 4) (Farzana et al., 2018).

According to Dutta et al. recycling waste batteries and electronics to recover nanomaterials has ushered in a new era in nanotechnology and environmental research (Dutta et al., 2018).

### Carbon Nanotubes

Carbon nanotubes (CNTs) synthesis from waste plastics is carried out in diverse systems, such as autoclaves, quartz tubes, crucibles, and muffle furnaces (Bazargan and McKay, 2012). Each method aims to disintegrate solid-state polymers into their carbon precursors via pyrolysis (Zhuo and Levendis, 2014). Polyolefin (~85.7wt % carbon content) is a polymeric material created from olefin monomer (C_n_H_2n_), and it is present in the large majority of plastic waste (Gong et al., 2012). Two waste polyolefins, polypropylene (PP) and polyethylene (PE) can be used for carbon materials synthesis. Other plastics integrated with additional elements such as polyvinyl alcohol (PVA) and polyethylene terephthalate (PET), can also be used for CNTs synthesis (Deng et al., 2016).

Gong et al. developed a new layer-by-layer assembling mechanism. In this method, waste PP was catalyzed via activated carbon and Ni_2_O_3_ in a quartz tube reactor. Activated carbon was effective in breaking PP into light hydrocarbons. Activated carbon also provided highly efficient catalytic conversion with Ni by encouraging dehydrogenation and aromatization with the formation of different aromatic groups. CNTs growth was accomplished based on benzene rings. The carboxylic moieties of activated carbon also enabled synergistic catalysis with Ni_2_O_3_. The highest carbon yield (~50 wt%) was reached at 820°C with a proportion of raw materials of PP:10Ni_2_O_3_:8AC (wt%) (Gong et al., 2012). Considering the high energy demand of this method, the recovery of Ni_2_O_3_ could be an alternative for more sustainable scale-up manufacturing.

Zhou et al. reported another combustion method for PE decomposition. This method generates light hydrocarbon *in-situ* during an exothermic process. This method utilized a stainless-steel wire mesh as a catalyst and a substrate. A ceramic filter was placed before the stainless-steel wire mesh to eliminate the soot that could deactivate the mesh that served as a catalyst. The yield of CNTs was higher than 10 wt% (Zhuo et al., 2010).

In another synthesis process, Zhang et al. mixed PP (~2 g), maleated PP (~0.5 g), and Ni catalysis powders (~0.5 g) in an autoclave. The resulting mixture was then heated for 12 h to a temperature of 700°C using an electric stove. It was then left to cool to ambient temperature. Carbon solid spheres were the only product obtained in absence of Ni from the reaction system which indicated that Ni powder catalyzed the decomposition of PP. MA-PP contributed to two actions in the growth process of CNTs, first improving the dispersion of Ni in PP, and then forming a homogenous system between carbon atoms and Ni catalysts. Ni particles were separated to form carbon-surrounded Ni particles and a high surface packing density of Ni particles enabled the nanotubes to grow along a consistent direction. This method of production of 160 nm CNTs had a yield of 80% (Zhang et al., 2008).

Bajad et al. attained approximately 45.8% yield in the production of MWNTs. These MWNTs were derived from PP waste catalyzed by Ni/Mo/MgO using a combustion technique (Bajad et al., 2015). The powdered catalysts and PP were placed in a covered silicon crucible and heated to 800°C in a muffle furnace. The Ni/Mo ratio was found to affect the yield and size of nanotubes. The HRTEM images exposed that the Ni/Mo ratio controlled both the yield and morphology of CNTs. Increasing the Mo content resulted in large diameter CNTs while lower Mo content gave higher yield with short-radius CNTs. The authors optimized the Ni/Mo mole ratio using response surface methodology (RSM) which proposed that 394% of carbon product could be yielded at a Ni/Mo mole ratio of 22.04. While 514% of CNTs would yield over Ni_4_Mo_0.2_MgO_1_ catalyst at 800°C, 5 g polymer weight, 150 mg catalyst weight, and combustion time of 10 min.

Table 4 summarizes the different methods used in the production of CNTs from plastic polymers commonly found in solid waste.

**Table 4:** Synthesis methods of CNTs from waste plastics.

**Waste plastics**	**Additives**	**Carbon yield (%)**	**References**
PP	Stainless steel 316 tube that acted as reactor and catalyst	42	Tripathi et al., 2017
PP and PE	Ni/Al–SBA-15 catalysts	74.1	Yang et al., 2016b
PP	Ni/Ca–Al catalyst	>10	Wu et al., 2012
LDPE and PP	Ni-Mo/Al_2_O_3_ catalyst	58	Aboul-Enein et al., 2018
PP	Ni/Mo/MgO catalyst	58	Song and Ji, 2011
HDPE and LDPE	Solvent free, cobalt acetate as catalyst	40	Pol and Thiyagarajan, 2010
PE	CuBr and NiO	NR	Gong et al., 2014a
HDPE	Stainless steel wire mesh	NR	Zhuo et al., 2010
PP	Maleated polypropylene and Ni as catalysts	NR	Zhang et al., 2008
PP	Ni/Mo/MgO	45.8	Bajad et al., 2015
PP	Organic-modified clay and supported Ni catalyst	41.16	Tang et al., 2005
Polyvinyl alcohol	Fly ash	NR	Nath and Sahajwalla, 2011a,b, 2012
PP	Ni catalyst	85	Mishra et al., 2012
PET	Catalysts free	~39	Berkmans et al., 2014

*NR, not reported in the manuscript*.

### Graphene

Graphene is a single-atom-thick sheet of sp2 hybridized carbon atoms packed into a honeycomb lattice structure. Prominent properties of this material are high surface area, high electrical conductivity, good chemical stability, and strong mechanical strength (Nair et al., 2008; Rao et al., 2009; Luo et al., 2012; Novoselov et al., 2012). Graphene revolutionized the health, energy, and environment sectors (Liu et al., 2013a; Quesnel et al., 2015; Surwade et al., 2015; Yang et al., 2016a).

Its electrical, optical, and mechanical properties (Bonaccorso et al., 2010; Soldano et al., 2010; Marinho et al., 2012) make graphene eligible for any application. There are two essential sources for the preparation of graphene: graphite and organic molecules (Strudwick et al., 2015). Typical methods for graphene preparation include the bottom-up approach, the top-down approach, epitaxial growth on silicon carbide (Mishra et al., 2016), mechanical cleavage (Bonaccorso et al., 2012), and chemical reduction of graphene oxide (Abdolhosseinzadeh et al., 2015). The bottom-up approach utilizes chemical vapor deposition (CVD) on metallic films (Strudwick et al., 2015). The top-down approach utilizes liquid exfoliation of graphite crystal (Coleman, 2013). Table 5 summarizes the recent approaches for graphene synthesis from waste materials.

**Table 5:** Synthesis of graphene from waste materials.

**Waste material**	**Fabrication technique**	**References**
PP	Catalytic carbonization at 700°C	Gong et al., 2014b
PTFE	Epitaxial growth on SiC	Manukyan et al., 2013
PS, grass blades, waste food, and grass,	CVD using Cu foil as catalyst	Ruan et al., 2011
PE and PS	CVD synthesis	Sharma et al., 2014
PET	Thermal decomposition in a closed system.	El Essawy et al., 2017
Graphite electrode of waste dry cell zinc–carbon batteries	Acid treatment then, Hammer method	Roy et al., 2016
PET, PE, PVC, PP, PS, and polymethylmethacrylate (PMMA)	Solid-state CVD	Cui et al., 2017
PET	Pyrolysis at 900 °C then catalytic graphitization at 2,400 °C followed by exfoliation	Ko et al., 2020

Graphene can be synthesized from various waste plastics using a variety of methods. Gong et al. were able to create high yields of graphene flakes. Their method used waste polypropylene (PP) catalyzed by organically modified montmorillonite. A uniform mixture of PP (~89 wt %), talcum (~11 wt %), and modified montmorillonite, was placed in a crucible and heated to 700°C for 15 min to obtain the carbonized char. After cooling the carbonized char was immersed in HF and HNO_3_. HF dissolved the impurities and HNO_3_ oxidized the amorphous carbon. After a repeated process of centrifuging and isolating from solution, graphene flakes were obtained (Gong et al., 2014b).

Manukyan et al. developed an energy-saving combustion method to prepare graphene sheets using waste polytetrafluoroethylene (PTFE) and silicon carbide (SiC). The process mechanism was similar to epitaxial growth on SiC, in which Si was removed by C_2_F_4_ through an exothermal reaction (Manukyan et al., 2013).

Ruan et al. developed a green synthesis method in which raw wastes including waste polystyrene, grass blades, waste food, and grass, were transformed into high-quality single-layered graphene. In this method, 10 mg of start materials were placed on a slightly bent piece of Cu foil held by a quartz boat in a CVD quartz tube. After low-pressure annealing at a temperature of approximately 1,050°C for 15 min in an inert atmosphere (Ar and H_2_), graphene growth was observed on the back of the Cu foil (Ruan et al., 2011).

Another CVD based graphene synthesis method used solid waste plastics roughly composed of 86% polyethylene and 14% polystyrene (Sharma et al., 2014). The method used two furnaces. In the first furnace, nearly 3 mg of plastic waste was put into a ceramic boat and kept at a temperature of approximately 500°C. In the second furnace, Cu foil was placed inside as substrate and kept at a temperature of approximately 1,020°C. Following pyrolysis, the degraded carbonaceous compounds were placed into the next furnace in a gas mixture (Ar/H_2_: 98/2.5 sc cm). The products then interacted with a Cu substrate causing graphene growth. In this method, the injection rate of disintegrated products was crucial for graphene crystal formation. Large hexagonal single-layered graphene was produced at a low rate of pyrolysis and injection. On the other hand, under a high injection rate, a bilayer or multilayer graphene was formed (Sharma et al., 2014).

## Environmental Applications of Waste-Derived Nanoparticles

The environmental applications of waste-derived nanoparticles will be discussed in this section and are presented in Table 6.

**Table 6:** Environmental applications of waste nanoparticles.

	**Nanoparticle**	**Source**	**Application**	**References**
Wastewater treatment and water remediation	Iron oxide nanoparticles	Mill scale	Dye removal (adsorption)	Arifin et al., 2017
	Magnetite (Fe_3_O_4_)	Iron ore tailings.	Dye removal (adsorption)	Giri et al., 2011
	Graphene	Polyethylene terephthalate (PET)	Dye removal (adsorption)	El Essawy et al., 2017
	CaCO_3_	Eggshell	Lead (Pb^2+^) adsorption	Wang et al., 2018
	Porous aerogels	Paper, cotton textiles, and plastic bottles	Oil adsorption	Thai et al., 2019
	NiFe_2_O_4_/ ZnCuCr-LDH composite	Saccharin wastewater	Dye removal (adsorption)	Zhang et al., 2020
	Silica nanoparticles (SiO_2_NPs)	Sugar cane waste ash	Dye removal (adsorption)	Rovani et al., 2018
	Silica nanoparticles (MW–nSiO_2_, nSiO_2_)	Corn husk waste	Dye removal (adsorption)	Peres et al., 2018
	Nnanocomposite of ZnO and CuO	Printed circuit boards	Dye removal (photocatalyst)	Nayak et al., 2019
	Metals-doped ZnO (M-ZnO)	Fabric filter dust	Dye removal (photocatalyst)	Wu et al., 2014
	Zero-valent iron nanoparticles	Pickling line of a steel plant	Nitrobenzene removal	Lee et al., 2015
Monitoring of pollutants in water	Carbon nanoparticles	Pomelo peels	Detection of mercury (Hg^2+^)	Lu et al., 2012
	Nano-cuprous oxide	Electrical waste	Detecting dopamine and mercury	Abdelbasir et al., 2018a
Capture of air pollutants	Porous silica nanoparticles (PSNs)	Rice husks	CO_2_ capture	Zeng and Bai, 2016

### Wastewater Treatment and Water Remediation

#### Adsorption

Industrial wastewater is contaminated with a mixture of pollutants unique to the industries that create them. For example, the textile industry produces waste streams contaminated with a variety of dyes. Water contaminated with these dyes must be treated to ensure public and environmental health (Arslan et al., 2016). Several research groups are attempting to find novel nanotechnology-enabled treatments for industrial wastewater using recycled nanoparticles, which helps meet circularity and sustainability goals. Arifin et al. (2017) developed iron oxide nanoparticles from mill scale and applied them to dye removal. In their process, iron oxide particles were removed from the unwanted components of mill scaling using magnetic separation techniques. The particles were then processed using conventional and high energy ball milling and treated with hexadecyltrimethylammonium bromide to prevent aggregation. In dye wastewater, the adsorption by the modified iron nanoparticles was above 99% with optimum adsorption of 99.93% with 53.76 nm particles (Arifin et al., 2017).

Giri et al. (2011) developed a method for creating magnetite (Fe_3_O_4_) nanoparticles from iron ore tailings. The formation of these particles was confirmed through powder X-ray diffraction (XRD), ultraviolet-visible spectrophotometry (UV–Vis) and Fourier-transform infrared spectroscopy (FT-IR) spectra. These particles were fast and effective in the removal of methylene blue and Congo red dyes. In optimal conditions, the monolayer adsorption capacities were 70.4 mg g^−1^ for methylene blue and 172.4 mg g^−1^ for Congo red. These nanoparticles compared well with particles created from reagent grade materials and as such could be a value-added product with possible applications in large scale wastewater treatment (Giri et al., 2011).

El Essawy et al. (2017) explored another means of removing dye from water using recycled Polyethylene terephthalate (PET) as a starting material for NP production. Through thermal dissociation, PET creates graphene. The graphene was characterized using SEM, TEM, Raman, BET, TGA, and FT-IR. The produced graphene showed high micropore volume and surface area. Potential for adsorption was assessed with methylene blue and acid blue 25 dyes. This graphene showed good adsorption of methylene blue with optimal adsorption at a pH of 12. The methylene blue dye reached equilibrium within 30 min. The adsorption of the acid blue 25 dye was optimal in acidic solutions and the adsorbed dye reached an equilibrium in approximately 50 min. The PET-based graphene showed effectiveness in removing both dyes from solutions (El Essawy et al., 2017).

Wang et al. developed engineered biochar functionalized with waste eggshell particles. The biochar is composed of three types of biomass pretreated with eggshell waste and prepared through slow pyrolysis. The eggshell particles are prepared using a method shown to make colloidal and nanosized eggshell particles (Hassan et al., 2014). Using characterization tools such as scanning electron microscopy eggshell particles were found on the surface and within the pore networks of the biochar. When studied the biochar treated with eggshells was more effective at lead (Pb^2+^) adsorption than pristine biochar due to the presence of CaCO_3_ from the eggshells (Wang et al., 2018).

Thai et al. (2019) reviewed the synthesis of highly porous aerogels from recycled materials such as paper, cotton textiles, and plastic bottles. Paper-derived aerogels were made through a simple process that required sonication of a solution of recycled cellulose with plyamide-epichlorohydrin, followed by freeze-drying at −98°C. Similarly, textile-derived aerogels were prepared by blending scraps of cotton cloth with DI water, followed by sonication in a solution of plyamide-epichlorohydrin. The resulting dispersion was freeze-dried at −98°C and cured at 120°C. Aerogels from recycled PET bottles were obtained by immersing PET fibers in a solution of NaOH heated at 80°C; the fibers were subsequently rinsed with DI water and then mixed with a solution of polyvinyl alcohol, glutaraldehyde, and HCl. Next, the mixture was sonicated, heated at 80°C, and freeze-dried. To confer superhydrophobicity to these aerogels, a coating of methyltrimethoxysilane was added at the end of the fabrication process. The ultra-low density and high absorption capacity of these waste-derived aerogels make them especially attractive for remediation applications such as the clean-up of oil spills in water bodies. For example, aerogels from cotton can achieve an absorption capacity above 100 grams of motor oil per gram of aerogel, which significantly outperforms most commercial sorbents (Thai et al., 2019).

Zhang et al. (2020) proposed and tested another method for treating waste dyewater using iron derived from the manufacturing of saccharin. In this process, NiFe_2_O_4_ nanoparticles that are used as a catalyst, are first extracted from the saccharin wastewater, which is challenging to treat. A mesoporous magnet NiFe_2_O_4_/ ZnCuCr-LDH composite is then created using a hydrothermal method. The created NiFe_2_O_4_/ ZnCuCr-LDH composites had a removal efficiency for Congo red of over 97% in cases where the initial concentration of the dye was between 100 and 450 mg l^−1^. The process treats the saccharin wastewater for iron contamination and reuses the waste iron to create a magnetic composite for the treatment of dye water (Zhang et al., 2020).

Rovani et al. and Peres et al. proposed methods of creating silicon nanoparticles from agricultural waste for the absorption of dyes. Rovani et al. created high purity silica nanoparticles (SiO2NPs) from sugar cane waste ash. The nanoparticles were tested for their capacity as absorbent material for the removal of acid orange 8 (AO8) dye. The silicon nanoparticles had an absorption capacity of 230 mg/g and the nanoparticles were able to be reused for up to five cycles (Rovani et al., 2018). Peres et al. synthesized silica nanoparticles from corn husk waste through a standard synthesis and a microwave synthesis method. These silicon nanoparticles were applied for the absorption of methyl blue dye. The microwave silica nanoparticles show higher values for surface area, poor volume, pore diameter, porosity, and purity compared to the traditionally synthesized silica nanoparticles. The microwave silica nanoparticles had an absorption capacity of 679.9 mg/g and a removal percentage of 80%. The thermodynamic results for the absorbent revealed a favorable spontaneous exothermic reaction for both nano-silica particles (Peres et al., 2018).

#### Photocatalytic Degradation

Nayak et al. (2019) synthesized a nanocomposite of ZnO and CuO using electronic waste, specifically printed circuit boards. These composites were formed by applying nitric acid to the memory slots and processing the leached liquid using an alkaline hydrothermal treatment. The composites were characterized using high-resolution transmission electron microscopy (HR-TEM), diffuse reflectance ultraviolet-visible spectrophotometry (UV-DRS), and UV–Vis. The nanocomposites were composed of CuO cores with ZnO nanostructures precipitated onto the cores. The nanoparticles proved to be effective photocatalyst for methyl orange dye degradation in the presence of visible light and hydrogen peroxide. These nanoparticles show promise as photo-Fenton catalysts for organic pollutants (Nayak et al., 2019).

Wu et al. explored another strategy for the photocatalytic degradation of pollutants in wastewater. Wu et al. prepared a metals-doped ZnO (M-ZnO) nanomaterial using fabric filter dust. The dopants used (Fe, Mg, Ca, and Al) and the zinc were all obtained from fabric filter dust without the addition of chemicals as elemental sources. The doped M-ZnO nanoparticles were prepared through sulfolysis combining co-precipitation processes. The nanoparticle acts as a favorable photocatalyst for the breakdown of organic substances, specifically phenol, under visible light irradiation (Wu et al., 2014).

#### Decomposition

Lee et al. studied the removal of nitrobenzene using synthesized zero-valent iron nanoparticles. The iron oxide to synthesize the nanoparticles comes from the pickling line of a steel plant. These particles are approximately 500 nm. The particles showed a reaction activity much higher than commercial zero-valent iron. Lee et al. proposed that in presence of the synthesized zero-valent iron nitrobenzene deoxidizes to nitrosobenzene. The nitrosobenzene is then reduced to aniline which is more biodegradable than nitrobenzene. When combined with biological processes these nanoparticles show promise in decomposing nitrobenzene in wastewater (Lee et al., 2015).

### Monitoring of Pollutants in Water

Laboratory tests utilized to identify pollutants in water are cost-prohibitive in some communities because they require trained personnel and expensive equipment to run. To address this problem, researchers are developing low-cost sensors to monitor pollutants using nanoparticles. Some researchers take it a step further using recycled nanoparticles. Lu et al. (2012) developed an optical sensor that uses recycled carbon nanoparticles from waste pomelo peels. Pomelo (*Citrus maximus*) has a grapefruit-like flavor with a much thicker peel and is a popular fruit from Brazil to Southeast Asia. The particles, derived from the hydrothermal processing of the pomelo peels, need no further chemical modification. The carbon particles have a reported quantum yield of approximately 6.9%. These particles were tested for use in the detection of mercury (Hg^2+^) based on the mercury-induced fluorescence quenching of the carbon particles. The particles' selectivity and sensitivity were excellent, and the particles had a limit of detection as low as 0.23 nM. The carbon nanoparticles have also been successful in mercury detection in lake water samples (Lu et al., 2012).

Abdelbasir et al. (2018a) developed an electrochemical sensor for environmental protection applications. The sensor was fabricated by anchoring copper nanoparticles to laser-scribed graphene electrodes. The nano-cuprous oxide was synthesized from electrical waste. The unique structure of the nanoparticles made them stable, linker-free, and size-tunable. The anchoring of the particles to the graphene electrode reduced internal/charge transfer resistance enhancing electrochemical performance. The effectiveness of the sensors in detecting dopamine and mercury was evaluated. In the case of dopamine, the sensor shows a linear calibration curve between 300 nM and 5 uM, a limit of detection of 200 nM, a sensitivity of 30 nA μM^−1^ cm^−2^, and a response time of 2.4 ± 0.7 s. In the case of mercury, the sensor shows a linear calibration curve between 0.02 and 2.5 ppm, a limit of detection of 25 ppb, a sensitivity of 10 nA ppm^−1^, and a response time of fewer than 3 min. The proposed approach presented to fabricate these sensors is scalable as wires are used in a variety of commonplace goods and industries (Abdelbasir et al., 2018a).

### Capture of Air Pollutants

Zeng et al. tested a means of deriving porous silica nanoparticles (PSNs) from rice husks with a simple template-free method. This method is inexpensive, fast, simple, and energy saving. (NH_4_)_2_SiF_6_ salt formed during the synthetic process serve as a porogen. Thus, providing control of the porosity of the PSNs without the need for extra pore-directing templates and post-heat-treatment by varying the molar ratios of HF/Si and NH4OH/Si. The PSNs were evaluated as support for polyethyleneimine (PEI). The PEI/silica composite reached an adsorption capacity of 159 mg/g at 75°C under 15% CO_2_. According to Zeng et al. this is superior to waste silica precursors reported in the literature. The sorbent also showed high stability during 20 cycles of absorption and desorption. This implies that it has potential in post combustions CO_2_ capture. The silica used could also be sourced from other waste streams such as bottom ash and fly ash (Zeng and Bai, 2016).

## Sustainability Considerations and Concluding Remarks

Throughout this review, different possibilities for using waste materials as inputs for the production of NPs have been exemplified and discussed in the context of research. While the concept of turning waste into advanced technologies for environmental applications may, at first glance, look like an attractive full-circle approach, there are significant knowledge gaps about engineered nanomaterials that should be addressed before transitioning these ideas from the lab to the real world; particularly regarding energy use, generation of secondary wastes, fate and transport behavior, exposure routes in different environments, and toxicity levels in diverse organisms (Kumar et al., 2014). As viable processes emerge from the proof-of-concept stage, careful cost-benefit analyses must be conducted. Though there is no simple path for overcoming the negative implications of the current “end-of-life” waste management systems, many experts have highlighted the importance of conducting life cycle assessments and risk analysis early on during the development of new technologies and processes, and also, to account for aging transformations and potential release of nanomaterials and byproducts in the environment. Thus, minimizing the potential for the future unveiling of unintended consequences (Dhingra et al., 2010; Grillo et al., 2015; Mitrano et al., 2015). However, performing the pertinent life cycle assessment and risk analyses implies having access to a sufficiently large body of data on emissions and environmental concentrations on engineered NPs. Hence, most data available in the literature have been obtained through modeling and simulation of the release of NPs from containing products during consumer manipulation; thus, empirical information on release coefficients throughout all stages of the life cycle (including production, usage, and disposal) is a limiting factor (Aitken et al., 2006; Gottschalk and Nowack, 2011).

At the production stage, the twelve principles of green chemistry provide a suitable framework for manufacturing NPs and NP-enabled technologies. These principles are intended to guide the design of chemical routes that yield enhanced sustainability outcomes (Gilbertson et al., 2015; Benelli, 2019). Nevertheless, the fact that NPs can be produced through sustainable approaches does not mean that the fabricated NPs are intrinsically safe and that their use and release in the environment will not cause harm. In the last decade, some studies have shown that engineered NPs that have already been used in different applications, including food manufacturing and packaging, could dramatically disrupt epigenetic mechanisms, and there are several questions regarding their ability to induce diseases (Smolkova et al., 2015). Perhaps, fullerenes are amongst the better-known class of engineered NPs. Several *in vitro* and *in vivo* studies have reported on the interactions and toxicity of carbon nanomaterials with various living systems (Liu et al., 2013b; Lalwani et al., 2016). Kagan et al. (2005) discussed how the very same properties that make CNTs so desirable for technological applications are also associated with inflammatory reactions and fibrogenic events that impair lung function in mice making their respiratory system more prone to infections (Kagan et al., 2005). The effects of inhalation of CNTs have been compared to those of asbestos fibers, which are known cytotoxic and carcinogenic particles (Sanchez et al., 2009).

In conclusion, to realize the potential benefits from the implementation of waste-derived NPs, a complex web of influencing factors must be understood, and decisions should err on the side of precaution and ethics. Scale and scope of the applications of these NPs, as well as strategies to enable closed-loop management of the engineered nanomaterials, should be defined on a regional basis and management policies should be informed by health and environmental science.

## Author Contributions

DV outlined and lead the drafting and editing of the manuscript. SA and KM performed an extensive literature search and contributed to the writing of sections and the construction of figures and tables. CL provided professional advice and participated in the revision of the final document. All authors read and approved the final manuscript.

## Conflict of Interest

The authors declare that the research was conducted in the absence of any commercial or financial relationships that could be construed as a potential conflict of interest.
